# Effects of Tai Chi Chuan on Inhibitory Control in Elderly Women: An fNIRS Study

**DOI:** 10.3389/fnhum.2019.00476

**Published:** 2020-01-22

**Authors:** Yuan Yang, Tingting Chen, Mingming Shao, Shoufu Yan, Guang H. Yue, Changhao Jiang

**Affiliations:** ^1^Beijing Key Laboratory of Physical Fitness Evaluation and Technical Analysis, Capital University of Physical Education and Sports, Beijing, China; ^2^The Center of Neuroscience and Sports, Capital University of Physical Education and Sports, Beijing, China; ^3^School of Kinesiology and Health, Capital University of Physical Education and Sports, Beijing, China; ^4^School of Education, Beijing Dance Academy, Beijing, China; ^5^Center for Mobility and Rehabilitation Engineering, Kessler Foundation, West Orange, NJ, United States; ^6^Rutgers New Jersey Medical School, Rutgers, The State University of New Jersey, Newark, NJ, United States

**Keywords:** inhibitory control, Tai Chi Chuan, elderly women, fNIRS (functional near-infrared spectroscopy), Flanker

## Abstract

**Background:**

Inhibitory control is a sub-ability of executive function and plays an important role in the entire cognitive process. However, declines in inhibitory control during aging significantly impair the quality of life of elderly people. Investigating methods to delay the decline of inhibitory control has become a focal point in current research. Tai Chi Chuan (TCC) is one effective method used to delay cognitive declines in older adults. However, the specific effects of TCC on inhibitory control and the mechanisms through which TCC may improve cognition in older adults have not been comprehensively investigated.

**Objective:**

The study explores possible neurological mechanisms related to the effects of TCC interventions on inhibitory control in older people using a functional near-infrared spectroscopy (fNIRS) technique and reaction times (RTs).

**Methods:**

A total of 26 healthy, elderly people who had not received TCC training completed all study procedures. The subjects were randomized to either the TCC group or the control group. Subjects in the TCC group were taught TCC by a certified instructor and trained for 8 weeks. The control group continued to perform general daily activities. The Flanker task was administered to every participant to evaluate inhibitory control pre- and post-intervention. While participants were performing the Flanker task, fNIRS data were collected.

**Results:**

Post-intervention, significant differences for incongruent flankers were found only for the TCC intervention group. Faster RTs were observed for the incongruent flankers in the TCC group than in the control group (*p* < 0.05). Analysis of the fNIRS data revealed an increase in oxy-Hb in the prefrontal cortex during the incongruent flankers after the TCC exercise intervention.

**Conclusion:**

The TCC intervention significantly improved inhibitory control in older adults, suggesting that TCC is an effective, suitable exercise for improving executive function and neurological health in elderly people.

**Clinical Trial Registration:**

Chinese Clinical Trial Register, ChiCTR1900028457.

## Introduction

The cognitive functioning of adults declines with advancing age. This decline increases the prevalence of cognitive impairment, and is currently a topic of focus in cognitive neuroscience research (Anderson and McConnell, 2007). Cognitive decline can reduce quality of life and social competence in the elderly population. Further, cognitive declines may result in adverse physiological and psychological consequences (Wu et al., 2013). Therefore, techniques to maintain the cognitive functioning of older people are of considerable significance and require further study. Maintenance strategies should focus on both physiological impact and psychological benefits.

Aerobic exercise is a type of endurance exercise that has aerobic metabolism as its primary component. Some researchers have utilized aerobic exercise as a method of preventing cognitive decline (Groot et al., 2016; Panza et al., 2018). However, existing studies have resulted in equivocal findings. A study investigating the effects of 30-min sessions of moderate-intensity, bicycle-based exercise found that the aerobic exercise improved cognitive function (Song and Yu, 2019). However, a 12-week intervention study, conducted by Kimura et al. (2010), did not reveal a significant change in the reaction times (RTs) or accuracy rate (AR) of older people in a switching task post-intervention. Furthermore, no evidence emerged from this study supporting the notion of exercise sessions improving cognitive functioning in older adults. These findings are, thus, inconsistent, potentially due to discrepant intervention methods. Therefore, it is important to investigate effective methods of maintaining cognitive function in older adults.

In recent years, researchers have utilized mind-body exercises to delay cognitive function declines in the elderly population (Lam et al., 2009, 2011; Fong et al., 2014; Zhang et al., 2014; Ikudome et al., 2016). Mind-body exercises are defined as aerobic exercises that improve balance and flexibility by concentrating on physical movements and controlling breathing. Such mind-body exercises include Tai Chi Chuan (TCC), Baduanjin, and Wuqinxi (Zheng et al., 2015). Mind-body exercises are known to improve the individual’s cardiopulmonary function as well as cognitive abilities (Miller and Taylor-Piliae, 2014). Nguyen and Kruse (2012) randomly assigned 102 healthy older participants to a TCC group or a control group. The TCC group trained for 6 months, whereas the control group maintained their normal daily activities. The cognitive functioning of the participants was tested using the Trail Making Test (TMT) at baseline and post-intervention. The results suggested that, after the TCC intervention, the older adults’ TMT scores were significantly improved. These findings support the hypothesis that long-term TCC exercise can improve cognitive functioning in older people.

Although mind-body exercises are beneficial to cognitive functioning in the elderly population, most current studies have focused on general cognitive functions. Few studies have examined the differential effects of mind-body exercise on specific cognitive functions. Research has demonstrated that the effect of exercise on executive functions is more significant than its effect on general cognitive functioning (Colcombe and Kramer, 2003). Executive function is an advanced cognitive function primarily characterized by inhibition, shifting, updating, and other cognitive subcomponents (Perner and Lang, 1999). Inhibitory control is a subclass of specific cognitive control functions and is defined as the ability to inhibit the activation of irrelevant information during cognitive processing, in this way, inhibitory control is important for all cognitive processes (Smith, 1999).

The Flanker task (Eriksen and Eriksen, 1974) is used to measure inhibitory control in older adults and requires the participant to identify a directional response to the central target stimulus presented between a series of lateral distractors (flankers). The flankers can be consistent with the central stimulus, representing the same directional response (< < < < <); alternatively, flankers can be inconsistent with the central stimulus, representing an incongruent, or opposite, directional response (> > < > >). Therefore, perception and responses must be allocated to the central target, for which processing of the flanking stimulus must be suppressed using inhibitory control. The suppression of response to the flanking stimuli is required to reduce the possibility of disturbance in the perceptually evoked response, while dominant perceptual cues activate the action schema (Amanda et al., 2019). In inconsistent arrays, where the target and flanking stimuli are mapped to opposite directional modes, there will be greater interference between the correct and incorrect responses. Thus, increased response inhibition is required to suppress the impact of interference. This is closely related to inhibitory control (Collette et al., 2009; Gothe et al., 2014; Chen K.C. et al., 2017; Guarino et al., 2019). Research has demonstrated that older people do not perform as well as younger people on inhibition tasks. This may be because older people are required to mobilize more cognitive resources to deal with conflicting information. This apparent decline implies that maintaining inhibitory control abilities with advancing age is critical (Zelazo et al., 2004; Kawai et al., 2012).

Prior studies on the cognitive benefits of mind-body exercises have not generally focused on neurological mechanisms, but those which did have primarily utilized electroencephalography (EEG) and functional magnetic resonance imaging (fMRI) (Chang et al., 2017; Tao et al., 2017b). Functional near-infrared spectroscopy (fNIRS) is a neuroimaging technique for investigating cortical hemodynamic responses (Chen T.T. et al., 2017). Since oxygenated hemoglobin (oxy-Hb) and deoxygenated hemoglobin (deoxy-Hb) have different absorption spectra in the infrared range, fNIRS imaging quantifies the levels of oxy-Hb and deoxy-Hb by evaluating changes in near-infrared light intensity migrating from the source to a detector. Signals reflecting changes in oxy-Hb and deoxy-Hb concentrations are calculated based on the modified Beer-Lambert’s law to infer local brain activation (Obrig and Villringer, 2003; Renata et al., 2019). The advantage of fNIRS is that the equipment is relatively stable, avoiding motion artifacts and making it suitable for the study of cortical responses during complex motor stimulation. Thus, fNIRS imaging can be used in sports and motion studies with high ecological validity. For example, the prefrontal cortex supports the cognitive control needed for complex motor learning and motor control, and data regarding activation of this area can be readily obtained using fNIRS (Leff et al., 2011). Due to these factors, fNIRS is more suitable than other imaging methods for the study of the cognitive benefits of sports (Tsujii and Watanabe, 2009).

To address the aforementioned knowledge gaps and equivocal research findings, we conducted a randomized controlled trial to investigate the effects of mind-body exercise (a TCC intervention) on specific cognitive functions in healthy older people and examined the relevant neurological mechanisms. Eight weeks of TCC exercises were performed and we employed the Flanker task as a cognitive experiment concomitant with fNIRS monitoring to evaluate whether the TCC exercises would significantly improve the inhibitory control of older adults. We hypothesized that 8 weeks of TCC practice could significantly modulate inhibitory control and improve neurological functioning in older adults.

## Methods

### Study Design and Participants

Participants were recruited from the local community in the Haidian District, Beijing, China. The eligibility criteria were being over the age of 60–75 and having a Mini-Mental State Examination score ≥24. The exclusion criteria were the presence of neurological conditions, depressive symptoms, or vision problems.

The participants were randomly assigned to either the TCC group or the control group. Participants in the experimental group learned TCC from a certified instructor and trained for 8 weeks (45 min per session for 3 days per week). Participants in the control group were instructed to maintain their original physical activity habits during the 8-week period. Each participant was administered the Flanker task (Eriksen and Eriksen, 1974) to evaluate their inhibitory control at baseline and post-intervention. The Flanker task was inspired by the classical paradigm of Eriksen and Eriksen (see section “Instruments”) (Eriksen and Eriksen, 1974). While performing the Flanker task, fNIRS data were collected from each participant. The primary outcome variable was changes in the oxy-Hb levels. The secondary outcome was the RTs for the incongruent flankers indicative of inhibitory control.

The Institutional Ethics Committee of Capital University of Physical Education and Sports approved the study. All participants signed an informed consent document prior to the intervention. A researcher who was not involved in the study used a serial number generator software program to randomly allocate the participants to the two groups.

### Intervention

**FIGURE 1:**
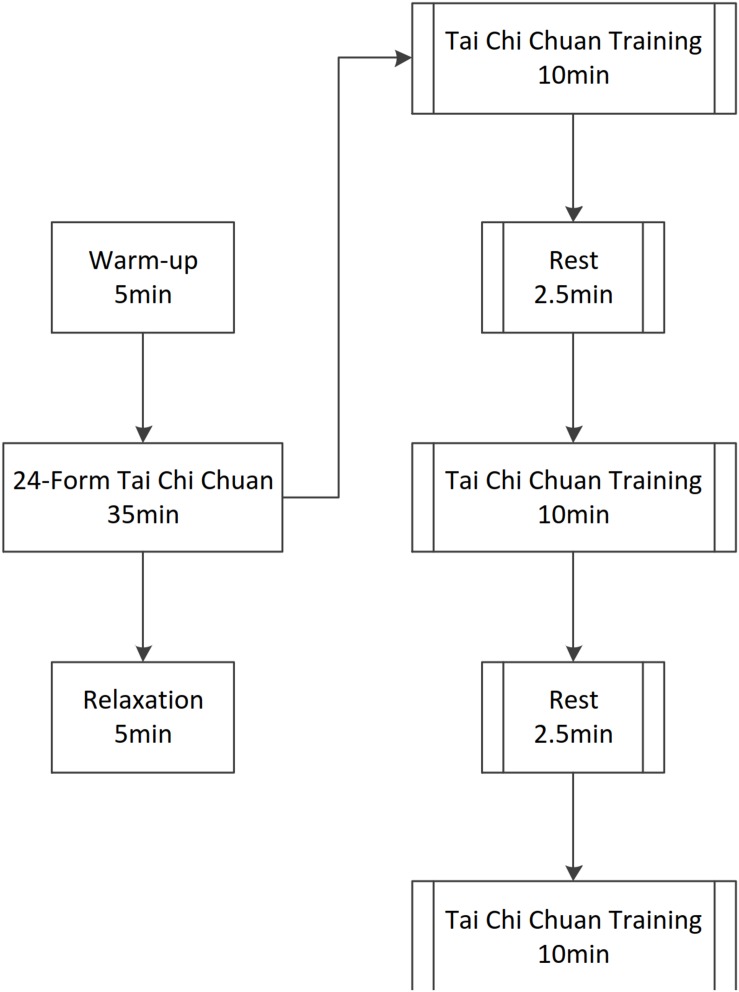
The TCC intervention lasted for 45 min per session. Each 45-min session included a 5-min warm-up, a 35-min TCC session, and a 5-min relaxation activity.

Before starting the 8-week TCC intervention (45 min per session for 3 days per week), the participants in the TCC group learned 24-form simplified TCC over six sessions (3 times per week for 2 weeks). Based on a compilation document from the General Administration of Sports of China, a number of studies on healthy elderly people have utilized 24-form simplified TCC (Chang et al., 2014). The TCC class was taught at a stadium in the Capital University of Physical Education and Sports by one instructor and two inspectors. After six classes, participants in the TCC group took part in the formal TCC intervention for 8 weeks. The formal TCC intervention comprised sessions of 45 min each, 3 days per week. Each 45-min session included a 5-min warm-up (muscle stretching), a 35-min TCC session, and a 5-min relaxation activity (breathing exercise). Participants were instructed to complete the Ratings of Perceived Exertion Scale after the TCC session to control the exercise intensity at a medium aerobic level. Participants in the control group did not undergo the TCC intervention and only performed their normal, general daily activities. The flow of the TCC exercise intervention program is shown in Figure 1.

To minimize confounding factors affecting cognitive function, the two groups were asked to maintain their normal daily lifestyle throughout the study and to inform the researchers of any lifestyle changes or emerging health-related events. In addition, all participants received weekly calls from a researcher involved in the study to monitor their health and any daily lifestyle changes, as well as to monitor their participation in other sports.

### Instruments

#### Neuroimaging

The results were assessed by experienced researchers at baseline and after the 8-week intervention. The researchers were blind to the participants’ group allocation. The primary outcome investigated was the fNIRS data, which included changes in oxy-Hb. The ETG-4000 system (Hitachi Ltd., Tokyo, Japan) was used for monitoring oxy-Hb levels. Participants’ oxy-Hb was monitored while performing the behavioral task. During monitoring, the light in the room was dimmed and the indoor environment was quiet. During the assessment, participants were asked to keep their posture stable and remain as still as possible. The fNIRS equipment was placed symmetrically over the frontal region of the participant’s head. The optical cap used consists of two measuring panels, each of which contains 22 channels. The arrangement of the optical poles is a 3 × 5 array and covers an area of 12 cm × 6 cm. The lowest detection point on the EEG 10–20 system was located at Fp1–Fp2. The absorption of near-infrared light was calculated using a time resolution of 0.1 s. In total, 44 channels of fNIRS data were obtained.

#### Cognitive Task

**FIGURE 2:**
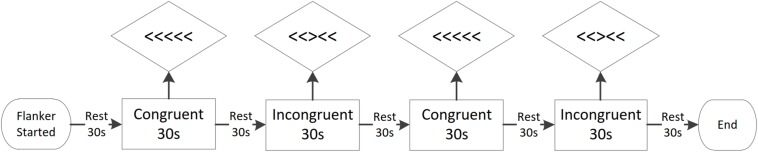
The full Flanker task consists of four blocks of 30 trials each, which include two congruent flanker tasks and two incongruent flanker tasks. The stimuli are randomly presented in the center of the computer screen. The stimuli include congruent flanker conditions (e.g., > > > > > and < < < < <) and incongruent flanker conditions (e.g., > > < > > and < < > < <) conditions. Each block has a 30 s rest period.

The second outcome studied was performance on the Flanker task. The classic arrow Flanker task paradigm was used to test inhibitory control. Before the assessment began, the participants were instructed to complete the training segment. The formal assessment began at the end of the training segment. The entire Flanker task consists of four blocks, which included two congruent flanker tasks and two incongruent flanker tasks. Each block comprised 30 trials. The stimulus was randomly presented in the center of the computer screen. The stimuli included congruent flanker conditions (e.g., > > > > > and < < < < <) and incongruent flanker conditions (e.g., > > < > > and < < > < <). In each block, the target marker (+) was first presented in the center of the screen, followed by the stimulus. Next, a blank screen was shown for up to 1,000 ms during the participant’s response window. The participants were required to react to the stimulus during the black screen time, after which the target marker appeared again and the next trial commenced. During the task, the background of the screen was black, and the stimuli were white. Blocks had a 30 s rest period between them. The participants were required to remain silent and to maintain a stable posture. The total time for the formal testing was approximately 5-min. During the test, the participants were required to judge the direction of the middle target arrow among the five arrows as quickly and accurately as possible. This required the participant to avoid false interference stimuli. Responses were recorded by choosing the correct key on the keypad (F key or J key representing left or right). After pressing the key, the computer recorded the participant’s AR and RTs. The Flanker task was conducted using E-Prime (version 2.0.10.182; Psychology Software Tools, Sharpsburg, PA, United States) on a 14-inch notebook computer with a 1,024 × 768 resolution screen. The E-Prime software was used to compile the Flanker task performance data. The Flanker task procedure is shown in Figure 2.

### Statistical Analysis

A mixed-model experiment was conducted with a 2 (group: TCC group vs. control group) × 2 (time: pre-test vs. post-test) × 2 (task: congruent vs. incongruent) design. The IBM SPSS software (version 23.0; IBM Corp., Armonk, NY, United States) was used for the statistical analysis. The RTs of the behavioral data were analyzed using repeated-measures analysis of variance (ANOVA) with a 2 (group: TCC group vs. control group) × 2 (time: pre-test vs. post-test) × 2 (task: congruent vs. incongruent) design. If there was a statistically significant interaction, a simple-effects analysis was used for further statistical analysis. The *p*-value was corrected with the Greenhouse-Geisser method, and the α significance level was set at 0.05.

**FIGURE 3:**
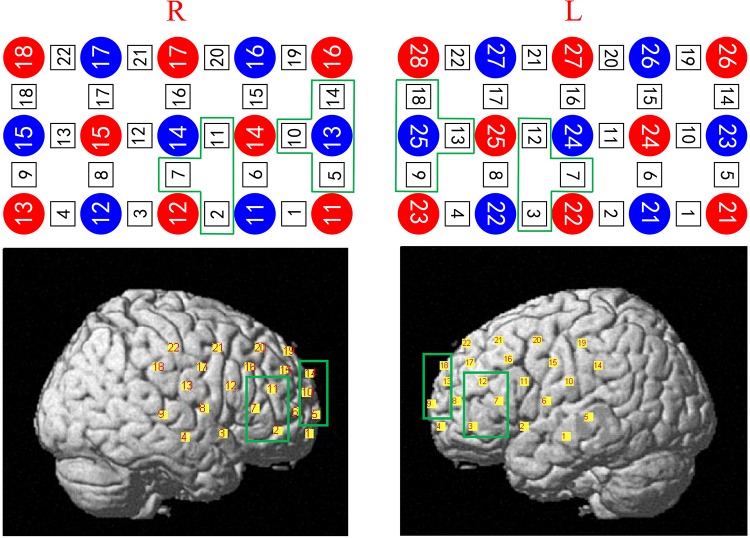
Four regions of interest (ROIs): the frontal superior left area (Frontal_Sup_L, which contains channels 9, 13, and 18), the frontal inferior left area (Frontal_Inf_L, which contains channels 3, 7, and 12), the frontal superior right area (Frontal_Sup_R, which contains channels 5, 10, and 14), and the frontal inferior area right (Frontal_Inf_R, which contains channels 2, 7, and 11) were classified according to the existing anatomical calibration system, and related research results. L: **left**. R: **right**.

We selected the frontal and temporal points of the brain because the fNIRS (ETG-4000 system) can only cover these two regions. Previous studies have shown that, as a key region of the cognitive control network, the dorsolateral prefrontal cortex (DLPFC) plays an important role in cognitive control processes (Miller and Cohen, 2001; Tao et al., 2017a). Thus, this choice of points was suitable. Four regions of interest (ROIs) were identified (Kameyama et al., 2004; Suto et al., 2004): the frontal superior left area (Frontal_Sup_L, which contained channels 9, 13, and 18), the frontal inferior left area (Frontal_Inf_L, which contained channels 3, 7, and 12), the frontal superior right area (Frontal_Sup_R, which contained channels 5, 10, and 14), and the frontal inferior area right (Frontal_Inf_R, which contained channels 2, 7, and 11). The ROIs were classified according to the existing anatomical calibration system, the Automated Anatomical Labeling software (Tzourio-Mazoyer et al., 2002), and related research results (Figure 3).

The data from the fNIRS were pre-processed by the xTopo software (version 2.08; Hitachi Ltd., Tokyo, Japan and Xu Cui, Stanford, CA, United States). The components with frequencies of less than 0.04 Hertz and more than 0.50 Hertz were filtered out. This decision was based on previous studies (Yanagisawa et al., 2010; Byun et al., 2014). Baseline correction was performed using a linear fitting function (Zhang et al., 2000). To this end, a linear fit was performed in the 10 s baseline before all of the active task segments and during the post-task baseline. The post-task baseline was determined as the average over the last 10 s of the resting period between active blocks (Ehlis et al., 2016). The mean values of the oxy-Hb signals of the four ROIs were calculated by averaging the same-condition blocks for each participant under each channel and then averaging the channels of the ROIs. The mean values of the oxy-Hb signals were analyzed using a repeated-measures ANOVA as a 2 (time: pre-test vs. post-test) × 2 (group: TCC group vs. control group) × 2 (task: congruent vs. incongruent) × 4 (ROI: Frontal_Sup_L vs. Frontal_Inf_L vs. Frontal_Sup_R vs. Frontal_Inf_R) design.

## Results

A total of 30 healthy older people who had not previously received TCC training participated in this study (mean age = 66.12 ± 3.81 years). Of the 30 participants, 26 (13 in the TCC group, 13 in the control group) completed all study procedures. Two participants dropped out of the TCC group owing to scheduling conflicts and two participants dropped out of the control group, one owing to an inability to perform to the Flanker task and one owing to scheduling conflicts.

### Demographic Data

Before the intervention, the demographic variables of the two groups were analyzed (Table 1). There were no significant differences (*p* > 0.05) between the groups in terms of age, height, weight, years of education, Mini-Mental State Examination score, average exercise levels (days per week), or body mass index. These findings indicated that the demographic characteristics of the two groups were sufficiently homogeneous.

**TABLE 1:** Demographic variables and MMSE scores (M ± SD, *n* = 26).

**Factor**	**TCC Group**	**Control Group**	***t***	***p***
Age (years)	66.31 ± 4.25	65.92 ± 3.48	0.25	0.80
Height (m)	1.61 ± 0.06	1.60 ± 0.05	0.39	0.70
Weight (kg)	62.15 ± 7.65	64.38 ± 7.57	−0.75	0.46
BMI	23.91 ± 2.88	25.02 ± 2.80	−0.99	0.33
Education (years)	13.46 ± 2.11	13.77 ± 2.17	−0.37	0.72
Exercise time (days/week)	3.69 ± 0.75	3.77 ± 0.83	−0.28	0.81
MMSE score	26.73 ± 2.63	27.47 ± 1.69	−0.91	0.37

### Homogeneity Test on the Baseline Flanker Task Scores

A homogeneity test was conducted on the baseline (pre-intervention) Flanker task scores for the TCC and control groups. The selected significance level was 0.05 (Table 2). The baseline flanker task scores did not differ significantly between the control group and the TCC group for the congruent flankers (*F*(1,24) = 0.001, *p* = 0.98 > 0.05) or incongruent flankers (*F*(1,24) = 0.78, *p* = 0.39 > 0.05).

**TABLE 2:** Significance analysis of the Flanker task pre-intervention.

	**Congruent**		**Incongruent**	
	***F***	***p***	***F***	***p***
Group	0.001	0.98	0.78	0.39

### Flanker Task RTs

**FIGURE 4:**
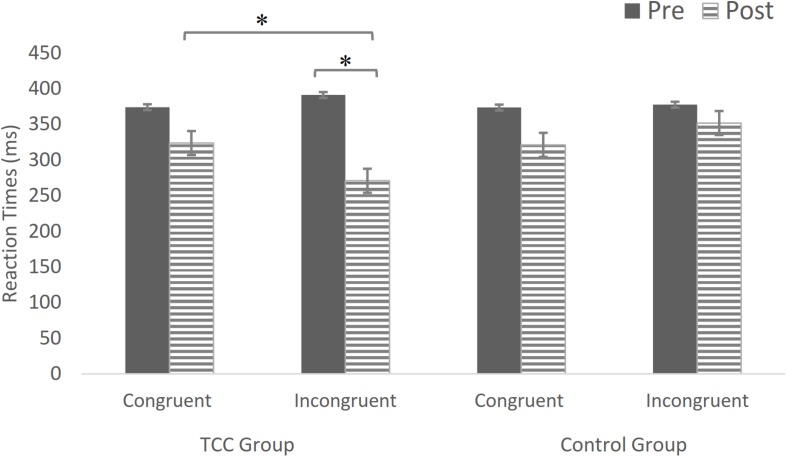
In the incongruent flanker task for the TCC group, the RTs at post-test was faster than those at pre-test. The difference was statistically significant. The incongruent flanker task RTs for the TCC group post-intervention were significantly faster than for the control group. There was no statistically significant change in the control group. ^∗^*p* < 0.05, significant difference; Bars indicate standard errors.

The Flanker task RTs were analyzed using a repeated-measures ANOVA (Table 3 and Figure 4) with a 2 (group: TCC group vs. control group) × 2 (time: pre-test vs. post-test) × 2 (task: congruent vs. incongruent) design. The interaction of time by group was not statistically significant for congruent flankers (*F*(1,24) = 0.01, *p* = 0.92 > 0.05). The main effect of time was statistically significant for incongruent flankers (*F*(1,24) = 91.03, *p* < 0.05). The main effect of group was statistically significant (*F*(1,24) = 4.91, *p* = 0.04 < 0.05) and the interaction of time by group was statistically significant (*F*(1,24) = 38.25, *p* < 0.05).

**TABLE 3:** RTs (ms) on the Flanker test (M ± SD).

**Task**	**Type**	**TCC Group**		**Control Group**	
		**Pre**	**Post**	**Pre**	**Post**
Flanker	Congruent	374.0 ± 59.0	323.7 ± 51.3	373.5 ± 45.9	321.2 ± 53.0
	Incongruent	391.1 ± 54.8	270.8 ± 30.0^∗^	377.5 ± 46.2	351.8 ± 38.9

*^∗^*p* < 0.05, significant difference.*

**FIGURE 5:**
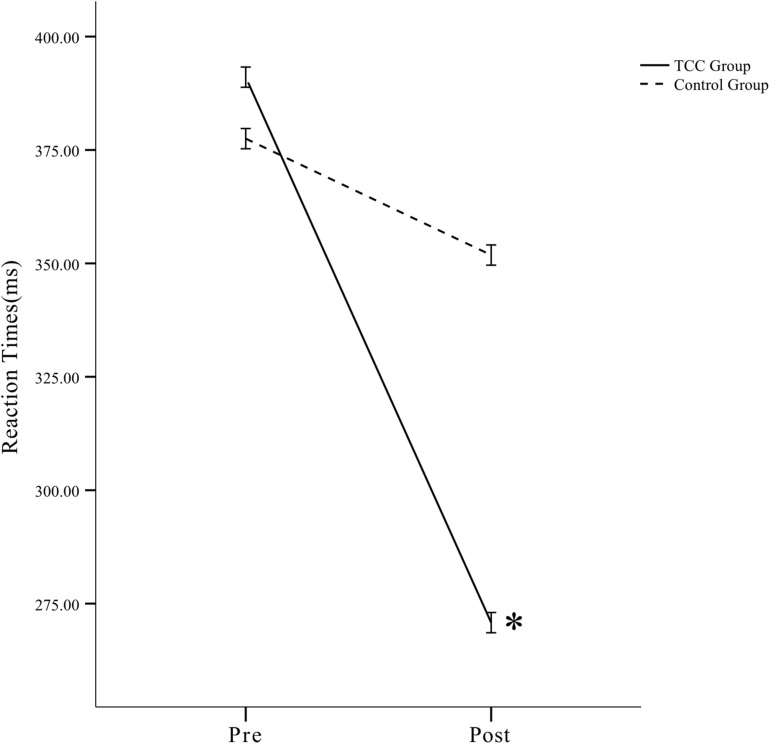
The RTs of the TCC and control groups in the incongruent task were lower than those before the intervention, whereas the reduction in the TCC group was significantly different. ^∗^*p* < 0.05, significant difference; Bars indicate standard errors.

Further, the simple-effects analysis showed that under incongruent task conditions, the TCC group was significantly different. The RTs at the post-test was faster than that at the pre-test, and the difference was significant (*p* < 0.05). There was no significant change in the control group (*p* > 0.05). The RTs in the TCC group when performing the incongruent task before the intervention was higher than that in the control group, but the difference was not significant (*p* > 0.05). The incongruent task RTs in the TCC group after the intervention was faster than that in the control group, and the difference was significant (*p* < 0.05) (Figure 5). Therefore, the TCC intervention had a positive impact on the inhibitory control ability of the elderly participants.

Figure 5 shows that, after the intervention, the RTs of the TCC and control groups in the incongruent flanker task were faster than those recorded before the intervention. The reduction in RTs in the TCC group was statistically significant.

### fNIRS Results

The oxy-Hb signal was analyzed using repeated-measures ANOVA with a 2 (time: pre-test vs. post-test) × 2 (group: TCC group vs. control group) × 2 (task: congruent vs. incongruent) × 4 (ROIs: Frontal_Sup_L vs. Frontal_Inf_L vs. Frontal_Sup_R vs. Frontal_Inf_R) design. The results showed that, in the incongruent flanker task, the main effect of time was statistically significant (*F*(1,24) = 4.683, *p* = 0.041 < 0.05). The interaction of time by group by ROI was also statistically significant (*F*(3,22) = 4.492, *p* = 0.012 < 0.05), which indicates that there were significant differences in the ROIs between the different groups pre- and post-intervention. The oxy-Hb signals in ROIs under different task conditions pre- and post-intervention are presented in Tables 4, 5.

**TABLE 4:** Oxy-Hb signal in ROIs under different task conditions pre-intervention (M ± SD).

**ROIs**	**TCC Group**		**Control Group**	
	**Congruent**	**Incongruent**	**Congruent**	**Incongruent**
Frontal_Sup_L	−0.013 ± 0.091	−0.013 ± 0.105	−0.028 ± 0.059	−0.013 ± 0.017
Frontal_Inf_L	−0.012 ± 0.107	0.003 ± 0.075	−0.024 ± 0.001	0.008 ± 0.003
Frontal_Sup_R	0.061 ± 1.902	−0.040 ± 0.044	0.091 ± 0.015	−0.024 ± 0.003
Frontal_Inf_R	−0.009 ± 0.010	−0.002 ± 0.778	−0.009 ± 0.001	−0.007 ± 0.003

**TABLE 5:** Oxy-Hb signal in ROIs under different task conditions post-intervention (M ± SD).

**ROIs**	**TCC Group**		**Control Group**	
	**Congruent**	**Incongruent**	**Congruent**	**Incongruent**
Frontal_Sup_L	0.041 ± 0.021	0.116 ± 0.143^∗^	−0.032 ± 0.058	−0.003 ± 0.071
Frontal_Inf_L	0.049 ± 0.030	0.093 ± 0.114^∗^	−0.016 ± 0.080	0.002 ± 0.120
Frontal_Sup_R	0.041 ± 0.015	−0.050 ± 0.037	0.089 ± 0.106	−0.002 ± 0.540
Frontal_Inf_R	−0.003 ± 0.008	−0.006 ± 0.004	0.003 ± 0.087	−0.006 ± 0.098

*^∗^*p* < 0.05, significant difference.*

**FIGURE 6:**
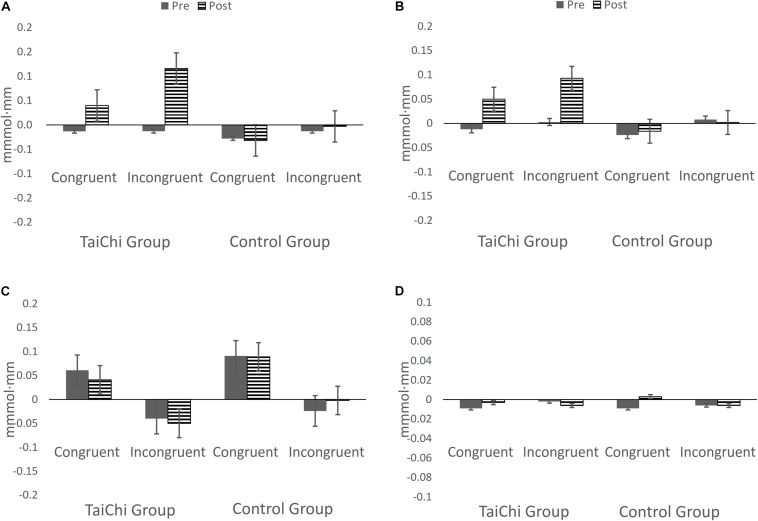
The oxy-Hb concentration change in ROIs during the incongruent flanker task. **(A)** The Frontal_Sup_L oxy-Hb signals for the TCC group performing the incongruent flanker task post-intervention were significantly higher than at pre-test. There was no statistically significant change for the control group. Post-intervention, the oxy-Hb signals for the TCC group were higher than for the control group. **(B)** The Frontal_Inf_L oxy-Hb signals for the TCC group were higher than that at pre-test when performing the incongruent flanker task post-intervention. There was no significant change for the control group. Post-intervention, the oxy-Hb signals for the TCC group were higher than for the control group and the difference was marginally significant. **(C)** The changes in the Frontal_Sup_R were not statistically significant. **(D)** The changes in the Frontal_Inf_R were not statistically significant. Bars indicate standard errors.

A simple-effects analysis was conducted on the three-way interaction of time by group by ROIs to explore the effects of the TCC intervention on the different ROIs in the two groups of participants. The results showed that the Frontal_Sup_L oxy-Hb signal in the TCC group was higher at post-test than at pre-test for the incongruent flanker task, and that there was a statistically significant difference (*p* < 0.05). However, there was no significant difference in the control group (*p* > 0.05). There was no significant difference in the oxy-Hb signal between the two groups pre-intervention (*p* > 0.05). Post-intervention, the oxy-Hb signal in the TCC group was higher than that in the control group (*p* < 0.05). The Frontal_Inf_L oxy-Hb signal in the TCC group was higher than that at pre-test when performing the incongruent flanker task post-intervention (*p* < 0.05). However, there was no statistically significant change in the control group (*p* > 0.05). There were no significant difference in the oxy-Hb signal between the two groups pre-intervention (*p* > 0.05). Post-intervention, the oxy-Hb signal in the TCC group was higher than that in the control group and the difference was borderline significant (*p* = 0.057). The changes in the other ROIs were not statistically significant (Figure 6).

## Discussion

Eight weeks of a TCC intervention can improve older people’s performance on the Flanker task and enhance brain activation in the left frontal lobe during an inhibition task (incongruent flankers). This indicates that TCC can improve the inhibitory control of older adults and enhance the activation of the brain regions related to inhibition. The results from the Flanker task showed that, in the incongruent flanker task, the RTs of the TCC group post-intervention were significantly faster than those pre-intervention, while the changes for the control group were not statistically significant. Post-intervention, the RTs for the TCC group were faster than those of the control group, and there were statistically significant differences. This finding suggests that the TCC exercise program improved Flanker task performance in older people. This result is consistent with those of previous studies. Ji et al. (2017) used a conflict control task to investigate the effects of different exercises on the executive function of older adults. Eighty-four, healthy, older participants were randomly divided into a TCC group, a fast walking group, and a control group. The participants were tested pre- and post-intervention, and the results showed that the test scores of the exercise group participants were significantly higher than those in the control group. In addition, the scores for the TCC group were significantly higher than those for the control group, and the ARs and RTs of the TCC group were higher than those of the fast walking group. Ji et al.’s (2017) findings indicate that their TCC intervention had a stronger positive effect on inhibition control in older people.

The Flanker task involves a cognitive component used in suppressing conflicting information (Kopp et al., 1996). The results of this study showed that, pre-intervention, the RTs on the incongruent flanker task were higher than in the congruent flanker task. With the increase in task difficulty, the RTs became slower. This finding indicates that participants were required to mobilize more cognitive resources to deal with more complex conflicting information. The results of this study confirm that these cognitive functions can be improved through using TCC exercises.

TCC is a mind-body exercise that integrates flexibility and coordination. Its spatial orientation changes greatly. The mental state of the participant undertaking TCC is concentrated, but relaxed, and the technique itself is closely related to restraint and control. The practice of TCC requires the integration of mental concentration and breathing control into physical motions to achieve a harmonious balance between body and mind (Lan et al., 2004, 2013). This combination produces psychological benefits, including improvements in attentiveness and reductions in stress and anxiety (Zhou et al., 2018). Moreover, TCC involves visual information processing that occurs simultaneously with the physical movements. This visual processing may improve the information processing abilities related to tasks involving cognitive processing and activities. The spatial orientation and action of TCC practice changes greatly. Elderly people frequently deal with the conflicting information as their spatial orientations and action directions are inconsistent when they perform the different exercises during TCC, and they are required to select the correct action. This physical process requires the individual to perform cognitive activities, including motor recall and task switching. Further, TCC involves the integration of the motor system and cognitive nervous system through two components: aerobic exercise and cognitive training. Previous studies have shown that both aerobic exercise and cognitive training can improve cognitive performance (Chang et al., 2010). Therefore, TCC can improve older people’s ability to deal with conflicting information as this study demonstrated.

The fNIRS results demonstrated that, compared with the control group, the participants who received 8 weeks of the TCC intervention had higher oxy-Hb concentrations in the Frontal_Inf_L and Frontal_Sup_L when they completed the Flanker task. The increase in oxy-Hb concentration reflects an increase in regional cerebral blood flow caused by the activation of cortical neurons. This finding suggests that the cortical activation was stronger in these areas (Byun et al., 2014). Therefore, TCC exercise evidently enhanced the activation of the left frontal lobe during the Flanker task. This finding is consistent with the conclusions of previous research. For example, Wei et al. (2013) used fMRI to compare the brain structures of 40 older people who had long-term experience practicing TCC in comparison to participants who did not practice TCC. The researchers found that the middle frontal sulcus and other brain regions were significantly thicker in the participants who practiced TCC long-term. Their results demonstrate that the practice of TCC long-term can result in changes to the local structures of the brain, and these changes are reflected in better executive control ability.

The frontal lobe is an important brain region for controlling executive function. Research has found that mind-body exercises can enhance activation in the frontal lobe when completing control tasks. Chen T.T. et al. (2017) used the Flanker task to examine the effect of a mind-body exercise intervention on executive control. The researchers found that 8 weeks of mind-body exercises enhanced activation in the prefrontal lobe and as well as executive functions. The frontal lobe is also associated with inhibitory control, which is strongly susceptible to brain aging. Compared with younger people, the activation of the frontal lobe differs in older people when they undergo the Flanker task (Zhu et al., 2010). Since frontal lobe dysfunction is associated with multiple diseases, strengthening activation through training can enhance self-regulation and used as a tool for treating, or preventing, related diseases. The results of this study are helpful in understanding how mind-body exercises influence brain activation and behavioral performance. The findings of this, and other, research suggest that mind-body exercises may induce these effects over time and are a useful tool for disease prevention and treatment.

After 8 weeks of TCC intervention, we found that activation in the left frontal lobe was significantly altered, but no changes were found in other brain regions. One possible explanation is that the spatial resolution of the fNIRS was limited. The spectral probe used in this study covered only the prefrontal and temporal lobes. Important subcortical areas, such as the hippocampus, are difficult to detect. In addition, different TCC exercise programs may lead to the activation of different brain regions. Therefore, these differences should be examined in future studies. There were also specific methodological limitations in this study. First, since all our participants were older women, the conclusions of this study may not reflect the changes experienced by older men. Second, the sample size was limited, thus affecting the statistical analyses. Sample sizes should be increased in future research.

## Conclusion

Tai Chi Chuan has a positive impact on the inhibitory control and regional brain activation of older adults. This was indicated by improved Flanker task performance and enhanced activation of the left frontal lobe-related brain areas in participants undergoing an 8-week TCC intervention. Tai Chi Chuan is easy to learn and a safe exercise, even for older adults. The results of this study are instructive for designing exercise programs for older adults.

## Data Availability Statement

The raw data supporting the conclusions of this article will be made available by the authors, without undue reservation, to any qualified researcher.

## Ethics Statement

The experiments were approved by the Capital University of Physical Education and Sports. All participants gave written informed consent in accordance with the Declaration of Helsinki. The experiments comply with the current laws of the country in which they were performed.

## Author Contributions

YY, SY, and CJ: conceptualization and formal analysis. YY, SY, TC, GY, and CJ: writing – review and editing. MS: software. CJ: resources, project administration, and funding acquisition. YY and MS: data curation. YY: writing – original draft preparation. TC: fNIRS data analysis.

## Conflict of Interest

The authors declare that the research was conducted in the absence of any commercial or financial relationships that could be construed as a potential conflict of interest.

## Abbreviations

ANOVA: analysis of variance
EEG: electroencephalography
fMRI: functional magnetic resonance imaging
fNIRS: functional near-infrared spectroscopy
Oxy-Hb: oxygenated hemoglobin
ROIs: regions of interest
RTs: reaction times.

## References

[B1] Amanda L. M. Abigail C. B. Jennifer L. S. Savage K. M. P. Tracey C. Matthew B. P. (2019). Acute and protracted disruptions to inhibitory control following sports related concussion. *Neuropsychologia* 131 223–232. 10.1016/j.neuropsychologia.2019.05.026 31152752

[B2] Anderson L. A. McConnell S. R. (2007). Cognitive health: an emerging public health issue. *Alzheimers Dement.* 3 s70–s73. 10.1016/j.jalz.2007.01.018 19595979

[B3] Byun K. Hyodo K. Suwabe K. Ochi G. Sakairi Y. Kato M. (2014). Positive effect of acute mild exercise on executive function via arousal-related prefrontal activations: an fNIRS study. *Neuroimage* 98 336–345. 10.1016/j.neuroimage.2014.04.067 24799137

[B4] Chang Y. K. Chu I. H. Liu J. H. Wu C. H. Chu C. H. Yang K. T. (2017). Exercise modality is differentially associated with neurocognition in older adults. *Neural Plast.* 2017 1–11. 10.1155/2017/3480413 28503331 PMC5414588

[B5] Chang Y. K. Nien Y. H. Chen A. G. Yan J. (2014). Tai ji quan, the brain, and cognition in older adults. *J. Sport Health Sci.* 3 36–42. 10.1016/j.jshs.2013.09.003

[B6] Chang Y. K. Nien Y. H. Tsai C. L. Etnier J. L. (2010). Physical activity and cognition in older adults: the potential of Tai chi chuan. *J. Aging Phys. Act.* 18 451–472. 10.1123/japa.18.4.451 20956845

[B7] Chen K. C. Weng C. Y. Hsiao S. Tsao W. L. Koo M. (2017). Cognitive decline and slower reaction time in elderly individuals with mild cognitive impairment. *Psychogeriatrics* 17 364–370. 10.1111/psyg.12247 28261945

[B8] Chen T. T. Yue G. H. Tian Y. X. Jiang C. H. (2017). Baduanjin mind-body intervention improves the executive control function. *Front. Psychol.* 7:2015. 10.3389/fpsyg.2016.02015 28133453 PMC5233682

[B9] Colcombe S. Kramer A. F. (2003). Fitness effects on the cognitive function of older adults: a meta-analytic study. *Psychol. Sci.* 14 125–130. 10.1177/1745691617707316 12661673

[B10] Collette F. Schmidt C. Scherrer C. Adam S. Salmon E. (2009). Specificity of inhibitory deficits in normal aging and Alzheimer’s disease. *Neurobiol. Aging* 30 875–889. 10.1016/j.neurobiolaging.2007.09.007 18029058

[B11] Ehlis A. C. Haeussinger F. B. Gastel A. Fallgatter A. J. Plewnia C. (2016). Task-dependent and polarity-specific effects of prefrontal transcranial direct current stimulation on cortical activation during word fluency. *Neuroimage* 140 134–140. 10.1016/j.neuroimage.2015.12.047 26748077

[B12] Eriksen B. A. Eriksen C. W. (1974). Effects of noise letters upon the identification of a target letter in a nonsearch task. *Percept. Psychophys.* 16 143–149. 10.3758/BF03203267

[B13] Fong D. Y. Chi L. K. Li F. Chang Y. K. (2014). The benefits of endurance exercise and Tai Chi Chuan for the task-switching aspect of executive function in older adults: an ERP study. *Front. Aging Neurosci.* 6:295. 10.3389/fnagi.2014.00295 25389403 PMC4211410

[B14] Gothe N. P. Fanning J. Awick E. Chung D. Wójcicki T. R. Olson E. A. (2014). Executive function processes predict mobility outcomes in older adults. *J. Am. Geriatr. Soc.* 62 285–290. 10.1111/jgs.12654 24521364 PMC3927159

[B15] Groot C. Hooghiemstra A. M. Raijmakers P. G. Berckel B. N. Scheltens P. Scherder E. J. (2016). The effect of physical activity on cognitive function in patients with dementia: a meta-analysis of randomized control trials. *Ageing Res. Rev.* 25 13–23. 10.1016/j.arr.2015.11.005 26607411

[B16] Guarino A. Favieri F. Boncompagni I. (2019). Executive functions in Alzheimer disease: a systematic review. *Front. Aging Neurosci.* 10:437. 10.3389/fnagi.2018.00437 30697157 PMC6341024

[B17] Ikudome S. Mori S. Unenaka S. Kawanishi M. Kitamura T. Nakamoto H. (2016). Effect of long-term body-mass-based resistance exercise on cognitive function in elderly people. *J. Appl. Gerontol.* 36 1519–1533. 10.1177/0733464815625834 26912733

[B18] Ji Z. Li A. Feng T. Liu X. You Y. Meng F. (2017). The benefits of tai chi and brisk walking for cognitive function and fitness in older adults. *PeerJ* 5:e3943. 10.7717/peerj.3943 29062610 PMC5652256

[B19] Kameyama M. Fukuda M. Uehara T. Mikuni M. (2004). Sex and age dependencies of cerebral blood volume changes during cognitive activation: a multichannel near-infrared spectroscopy study. *Neuroimage* 22 1715–1721. 10.1016/j.neuroimage.2004.03.050 15275927

[B20] Kawai N. Kubo-Kawai N. Kubo K. Terazawa T. Masataka N. (2012). Distinct aging effects for two types of inhibition in older adults: a near-infrared spectroscopy study on the Simon task and the flanker task. *Neuroreport* 23 819–824. 10.1097/WNR.0b013e3283578032 22828408

[B21] Kimura K. Obuchi S. Arai T. Nagasawa H. Shiba Y. Watanabe S. (2010). The influence of short-term strength training on health-related quality of life and executive cognitive function. *J. Physiol. Anthropol.* 29 95–101. 10.2114/jpa2.29.95 20558967

[B22] Kopp B. Rist F. Mattler U. (1996). N200 in the flanker task as a neurobehavioral tool for investigating executive control. *Psychophysiology* 33 282–294. 10.1111/j.1469-8986.1996.tb00425.x 8936397

[B23] Lam L. Tam C. Lui V. Chan W. Chan S. Chiu H. (2009). Modality of physical exercise and cognitive function in Hong Kong older Chinese community. *Int. J. Geriatr. Psychiatry* 24 48–53. 10.1002/gps.2072 18615844

[B24] Lam L. C. Chau R. C. Wong B. M. Fung A. W. Liu V. W. Tam C. C. (2011). Interim follow-up of a randomized controlled trial comparing Chinese style mind body (Tai Chi) and stretching exercises on cognitive function in subjects at risk of progressive cognitive decline. *Int. J. Geriatr. Psychiatry* 26 733–740. 10.1002/gps.2602 21495078

[B25] Lan C. Chen S. Y. Lai J. S. (2004). Relative exercise intensity of tai chi chuan is similar in different ages and gender. *Am. J. Chin. Med.* 32 151–160. 10.1142/S0192415X04001746 15154294

[B26] Lan C. Chen S. Y. Lai J. S. Wong M. K. (2013). Tai chi chuan in medicine and health promotion. *Evid. Based Complementary Altern. Med.* 2013 1–17. 10.1155/2013/502131 24159346 PMC3789446

[B27] Leff D. R. Orihuela-Espina F. Elwell C. E. Athanasiou T. Delpy D. T. Darzi A. W. (2011). Assessment of the cerebral cortex during motor task behaviours in adults: a systematic review of functional near infrared spectroscopy (fNIRS) studies. *Neuroimage* 54 2922–2936. 10.1016/j.neuroimage.2010.10.058 21029781

[B28] Miller E. K. Cohen J. D. (2001). An integrative theory of prefrontal cortex function. *Annu. Rev. Neurosci.* 24 167–202. 10.1146/annurev.neuro.24.1.167 11283309

[B29] Miller S. M. Taylor-Piliae R. E. (2014). Effects of tai chi on cognitive function in community-dwelling older adults: a review. *Geriatr. Nursing* 35 9–19. 10.1016/j.gerinurse.2013.10.013 24252560

[B30] Nguyen M. H. Kruse A. (2012). A randomized controlled trial of Tai chi for balance, sleep quality and cognitive performance in elderly Vietnamese. *Clin. Intervent. Aging* 7 185–190. 10.2147/CIA.S32600 22807627 PMC3396052

[B31] Obrig H. Villringer A. (2003). Beyond the visible - imaging the human brain with light. *J. Cereb. Blood Flow Metab.* 23 1–18. 10.1097/01.wcb.0000043472.45775.29 12500086

[B32] Panza G. A. Taylor B. A. Macdonald H. V. Johnson B. T. Zaleski A. L. Livingston J. (2018). Can exercise improve cognitive symptoms of Alzheimer’s disease? *J. Am. Geriatr. Soc.* 66 487–495. 10.1111/jgs.15241 29363108

[B33] Perner J. Lang B. (1999). Development of theory of mind and executive control. *Trends Cogn. Sci.* 3 337–344. 10.1016/S1364-6613(99)01362-5 10461196

[B34] Renata D. L. Laura P. Anna B. Chiara B. Yoko H. Yasuyo M. (2019). Recommendations for motion correction of infant fNIRS data applicable to multiple data sets and acquisition systems. *Neuroimage* 200 511–527. 10.1016/j.neuroimage.2019.06.056 31247300

[B35] Smith E. E. (1999). Storage and executive processes in the frontal lobes. *Science* 283 1657–1661. 10.1126/science.283.5408.1657 10073923

[B36] Song D. Yu D. S. F. (2019). Effects of a moderate-intensity aerobic exercise programme on the cognitive function and quality of life of community-dwelling elderly people with mild cognitive impairment: a randomised controlled trial. *Int. J. Nurs. Stud.* 93 97–105. 10.1016/j.ijnurstu.2019.02.019 30901716

[B37] Suto T. Fukuda M. Ito M. Uehara T. Mikuni M. (2004). Multichannel near-infrared spectroscopy in depression and schizophrenia: cognitive brain activation study. *Biol. Psychiatry* 55 501–511. 10.1016/j.biopsych.2003.09.008 15023578

[B38] Tao J. Chen X. Egorova N. Liu J. Xue X. Wang Q. (2017a). Tai chi chuan and baduanjin practice modulates functional connectivity of the cognitive control network in older adults. *Sci. Rep.* 7:41581. 10.1038/srep41581 28169310 PMC5294576

[B39] Tao J. Liu J. Liu W. Huang J. Kong J. (2017b). Tai chi chuan and Baduanjin increase grey matter volume in older adults: a brain imaging study. *J. Alzheimers Dis.* 60 1–12. 10.3233/JAD-170477 28869478 PMC5659386

[B40] Tsujii T. Watanabe S. (2009). Neural correlates of dual-task effect on belief-bias syllogistic reasoning: a near-infrared spectroscopy study. *Brain Res.* 1287 118–125. 10.1016/j.brainres.2009.06.080 19577547

[B41] Tzourio-Mazoyer N. Landeau B. Papathanassiou D. Crivello F. Etard O. Delcroix N. (2002). Automated anatomical labeling of activations in SPM using a macroscopic anatomical parcellation of the MNI MRI single-subject brain. *Neuroimage* 15 273–289. 10.1006/nimg.2001.0978 11771995

[B42] Wei G. X. Xu T. Fan F. M. Dong H. M. Jiang L. L. Li H. J. (2013). Can TaiChi reshape the brain? a brain morphometry study. *PLoS One* 8:e61038. 10.1371/journal.pone.0061038 23585869 PMC3621760

[B43] Wu Y. Wang Y. Burgess E. O. Wu J. (2013). The effects of Tai Chi exercise on cognitive function in older adults: a meta-analysis. *J. Sport Health Sci.* 2 193–203. 10.1016/j.jshs.2013.09.001

[B44] Yanagisawa H. Dan I. Tsuzuki D. Kato M. Okamoto M. Kyutoku Y. (2010). Acute moderate exercise elicits increased dorsolateral prefrontal activation and improves cognitive performance with stroop test. *Neuroimage* 50 1702–1710. 10.1016/j.neuroimage.2009.12.023 20006719

[B45] Zelazo P. D. Craik F. I. M. Booth L. (2004). Executive function across the life span. *Acta Psychol.* 115 167–183. 10.1016/j.actpsy.2003.12.005 14962399

[B46] Zhang R. Zuckerman J. H. Levine B. D. (2000). Spontaneous fluctuations in cerebral blood flow: insights from extended-duration recordings in humans. *Am. J. Physiol. Heart Circ. Physiol.* 278 H1848–H1855. 10.1046/j.1365-201X.2000.00727.x 10843881

[B47] Zhang X. Ni X. Chen P. (2014). Study about the effects of different fitness sports on cognitive function and emotion of the aged. *Cell Biochem. Biophys.* 70 1591–1596. 10.1007/s12013-014-0100-8 24997050

[B48] Zheng G. Liu F. Li S. Huang M. Tao J. Chen L. (2015). Tai chi and the protection of cognitive ability: a systematic review of prospective studies in healthy adults. *Am. J. Prev. Med.* 49 89–97. 10.1016/j.amepre.2015.01.002 26094229

[B49] Zhou M. Liao H. Sreepada L. P. Ladner J. R. Balschi J. A. Lin A. P. (2018). Tai chi improves brain metabolism and muscle energetics in older adults. *J. Neuroimaging* 28 359–364. 10.1111/jon.12515 29667260 PMC6055800

[B50] Zhu D. C. Zacks R. T. Slade J. M. (2010). Brain activation during interference resolution in young and older adults: an fMRI study. *Neuroimage* 50 810–817. 10.1016/j.neuroimage.2009.12.087 20045067 PMC2823923

